# Climate links leaf shape variation and functional strategies in quinoa’s wild ancestor

**DOI:** 10.1093/aobpla/plaf049

**Published:** 2025-09-05

**Authors:** Jonatan Rodriguez, Vilma B Quipildor, Eugenia M Giamminola, Sergio J Bramardi, David Jarvis, Jeff Maughan, Jiemeng Xu, Hafiz U Farooq, Pablo Ortega-Baes, Eric Jellen, Mark Tester, Daniel Bertero, Ramiro N Curti

**Affiliations:** Laboratorio de Investigaciones Botánicas (LABIBO), Facultad de Ciencias Naturales, Universidad Nacional de Salta and Consejo Nacional de Investigaciones Científicas y Técnicas (CONICET), Av. Bolivia 5150, CCT Salta-Jujuy 4400, Argentina; Laboratorio de Investigaciones Botánicas (LABIBO), Facultad de Ciencias Naturales, Universidad Nacional de Salta and Consejo Nacional de Investigaciones Científicas y Técnicas (CONICET), Av. Bolivia 5150, CCT Salta-Jujuy 4400, Argentina; Laboratorio de Investigaciones Botánicas (LABIBO), Facultad de Ciencias Naturales, Universidad Nacional de Salta and Consejo Nacional de Investigaciones Científicas y Técnicas (CONICET), Av. Bolivia 5150, CCT Salta-Jujuy 4400, Argentina; Centro de Investigaciones en Toxicología Ambiental y Agrobiotecnología del Comahue (CITAAC-CONICET), Universidad Nacional del Comahue, Buenos Aires 1400, Neuquén Q8300IBX, Argentina; Department of Plant and Wildlife Sciences, Brigham Young University (bYU), Provo UT 84602, United States; Department of Plant and Wildlife Sciences, Brigham Young University (bYU), Provo UT 84602, United States; Biological and Environmental Sciences and Engineering Division, King Abdullah University of Science and Technology (KAUST), Thuwal 23955, Saudi Arabia; Biological and Environmental Sciences and Engineering Division, King Abdullah University of Science and Technology (KAUST), Thuwal 23955, Saudi Arabia; Laboratorio de Investigaciones Botánicas (LABIBO), Facultad de Ciencias Naturales, Universidad Nacional de Salta and Consejo Nacional de Investigaciones Científicas y Técnicas (CONICET), Av. Bolivia 5150, CCT Salta-Jujuy 4400, Argentina; Department of Plant and Wildlife Sciences, Brigham Young University (bYU), Provo UT 84602, United States; Biological and Environmental Sciences and Engineering Division, King Abdullah University of Science and Technology (KAUST), Thuwal 23955, Saudi Arabia; Facultad de Agronomía, Universidad de Buenos Aires and Instituto de Investigaciones Fisiológicas y Ecológicas Vinculadas a la Agricultura (IFEVA-CONICET), C1053 Cdad. Autónoma de Buenos Aires, Buenos Aires, Argentina; Laboratorio de Investigaciones Botánicas (LABIBO), Facultad de Ciencias Naturales, Universidad Nacional de Salta and Consejo Nacional de Investigaciones Científicas y Técnicas (CONICET), Av. Bolivia 5150, CCT Salta-Jujuy 4400, Argentina

**Keywords:** climate adaptation, *Chenopodium hircinum;* common garden, functional–physiological traits, intraspecific variation, morphometric analysis

## Abstract

Understanding how leaf morphology mediates plant responses to environmental variability is critical for predicting species adaptability under climate change. This study examines whether intraspecific variation in leaf shape among *Chenopodium hircinum* populations is linked to physiological and functional trait differences and whether such variation reflects adaptive responses to source climate. We cultivated 11 populations of *C*. *hircinum* from diverse climatic origins in a common garden experiment. Leaf shape was quantified using descriptors (aspect ratio, circularity, solidity), landmarks, and Elliptical Fourier Descriptors. Physiological traits (stomatal conductance, leaf temperature, chlorophyll content) and functional traits (leaf area, leaf dry weight and leaf mass per area) were measured and analysed in relation to shape and environmental data. Leaf morphology varied significantly among populations and was associated with climatic conditions at origin, especially mean summer temperature. Functional and physiological traits were not directly correlated with environmental variables but showed strong associations with leaf shape. Landmark-based PC2 (lobed vs. rounded forms) and aspect ratio emerged as key predictors of trait variation. Most trait variation occurred at the individual level rather than among populations. Our findings highlight leaf shape as a central mediator linking environmental heterogeneity to physiological function. This suggests that morphology-driven trait integration may enhance adaptability in *C*. *hircinum*. Intraspecific diversity in shape and associated traits could serve as a reservoir of resilience under climate change, reinforcing the evolutionary and applied significance of wild relatives in crop improvement.

## Teaser

Leaves are plants’ primary interface with their environment, yet how their shapes influence function remains debated. We studied the wild ancestor of quinoa, Chenopodium hircinum, to investigate whether differences in leaf shape across populations are linked to climate and plant performance. Using a common garden experiment, we found that populations from warmer regions tended to produce leaves with distinct shapes, and these shapes were strongly associated with physiological and functional traits such as leaf temperature, stomatal conductance, and biomass investment. Surprisingly, the traits themselves were not directly related to climate, but were mediated through leaf shape. This highlights leaf morphology as a key integrator of environmental signals and plant function, suggesting that shape diversity may provide resilience to climate change and offer valuable traits for crop improvement.

## Introduction

The functional significance of leaf shape remains a central theme in plant evolutionary ecology and ecophysiology. As the primary photosynthetic organs of terrestrial plants, leaves are essential for individual survival and ecosystem functioning (Niklas et al. 2023). Understanding the relationships between leaf traits and environmental variables is increasingly important in the context of global climate change (Guerin et al. 2012). Given that variation in leaf shape and associated physiological traits reflects plant adaptation to diverse habitats, it is expected that specific leaf types will exhibit convergent patterns in functional traits and physiological processes in response to particular environmental conditions (Wright et al. 2004).

The diversity of angiosperm leaf shapes across latitudinal and altitudinal gradients suggests that environmental factors such as physical and biotic (i.e. herbivory) play a key role in shaping leaf morphology (Yang et al. 2015, Herrera 2017). Among these environmental factors, temperature has emerged as particularly influential in explaining observed patterns of variation. The strong associations between leaf shape, temperature, and physiological traits imply that shifts in selective regimes along climatic gradients can drive functional adaptation (Ferris 2019, Caruso et al. 2020). Several hypotheses have been proposed to explain these patterns, including thermoregulatory strategies and hydraulic constraints (Nicotra et al. 2011, Givnish and Kriebel 2017). However, temperature has not always been the primary explanatory factor associated with variation in leaf shape at the inter- and intraspecific levels (Royer et al. 2008, 2012, Campitelli et al. 2013, Campitelli and Stinchcombe 2013).

The Leaf Economic Spectrum (LES) offers a valuable framework for understanding trade-offs in leaf functional traits related to resource acquisition and conservation (Wright et al. 2004). The LES describes a continuum of strategies, from fast-return leaves, characterized by rapid resource acquisition and short lifespans, to slow-return leaves, which acquire resources more conservatively and exhibit greater longevity (Reich et al. 1997). This spectrum is strongly structured by correlations among leaf mass per area (LMA), photosynthetic and respiratory rates, and nutrient concentrations (Wright et al. 2004, Niklas et al. 2007). Climatic gradients also influence the expression of these traits, with certain environmental conditions favouring specific combinations of leaf form and function. For instance, arid environments often select for small, thick leaves with conservative traits such as high water-use efficiency—an expression of the LES ‘slow-return’ strategy typified by high LMA and long leaf lifespan (Reich et al. 1999, Wang et al. 2023). Conversely, in resource-rich environments like tropical rainforest understories or highly fertile riparian zones, plants often exhibit a contrasting ‘fast-return’ strategy. This involves large, thin leaves with a low LMA, high photosynthetic rates, and a short leaf lifespan. These traits enable rapid resource acquisition and carbon gain, reflecting a strategy of maximizing growth in conditions where water and nutrients are abundant and competition for light might be intense (Wright et al. 2004).

Previous studies investigating the relationship between leaf shape, functional traits, and environment have generally focused on broad morphological categories, such as entire versus dentate, or lobed versus unlobed leaves, and have primarily examined woody species, including trees, shrubs, and vines. However, recent evidence suggests that the LES framework can also be applied to herbaceous taxa, allowing for the exploration of shape–function relationships at both inter- and intraspecific levels (Mason and Donovan 2015, Giupponi 2020). Despite these advances, physiological trade-offs and the limited effect of temperature on certain traits indicate that observed patterns may diverge from theoretical expectations or be highly species-specific (Niklas et al. 2023, Everingham et al. 2024). Similar inconsistencies have been reported in other growth forms at the intraspecific level (Niinemets 2015).

Adding to this complexity, many herbaceous species exhibit considerable intraspecific variation in leaf shape that goes beyond binary classifications. In particular, species with lobed leaves often vary in lobe number, depth, position, and distribution along the lamina (Gupta et al. 2020, Balant et al. 2024, Hightower et al. 2024). This raises the question if intraspecific variation in leaf shape contributes to functional differentiation relevant to thermal adaptation?

The significance of herbaceous species closely related to crops, including their wild relatives, is heightened in the context of climate change (Maxted and Magos Brehm 2023). Their adaptive capacity under diverse environmental pressures provides a rich source of genetic variation for enhancing crop resilience (Renzi et al. 2022). Recent interest in *Chenopodium hircinum* Schrad (Amaranthaceae), the putative ancestor of quinoa (*Chenopodium quinoa* Willd.), stems from its importance as a wild relative genetic resource for improving crop resilience (Xu et al. 2025). In Argentina, *C*. *hircinum* populations inhabit a diverse range of environments, including some of the warmest regions of South America (Curti et al. 2023b). Moreover, recent studies have shown that variation in phenological and leaf shape traits among populations is associated with the environmental conditions at their origins, with temperature emerging as the most influential factor (Curti et al. 2023a, 2025).

In this study, we assessed whether population differentiation in the relationship between leaf shape and source climate among *C*. *hiricnum* populations also extends to physiological and functional traits. We hypothesize that leaf shape variation reflects adaptation to the thermal environment of origin and that this morphological variation is functionally significant. Accordingly, we expected to find associations between leaf shape and physiological performance, mediated by the environmental conditions of the populations’ native habitats.

## Materials and methods

### Population origins and sampling

**Figure 1.**
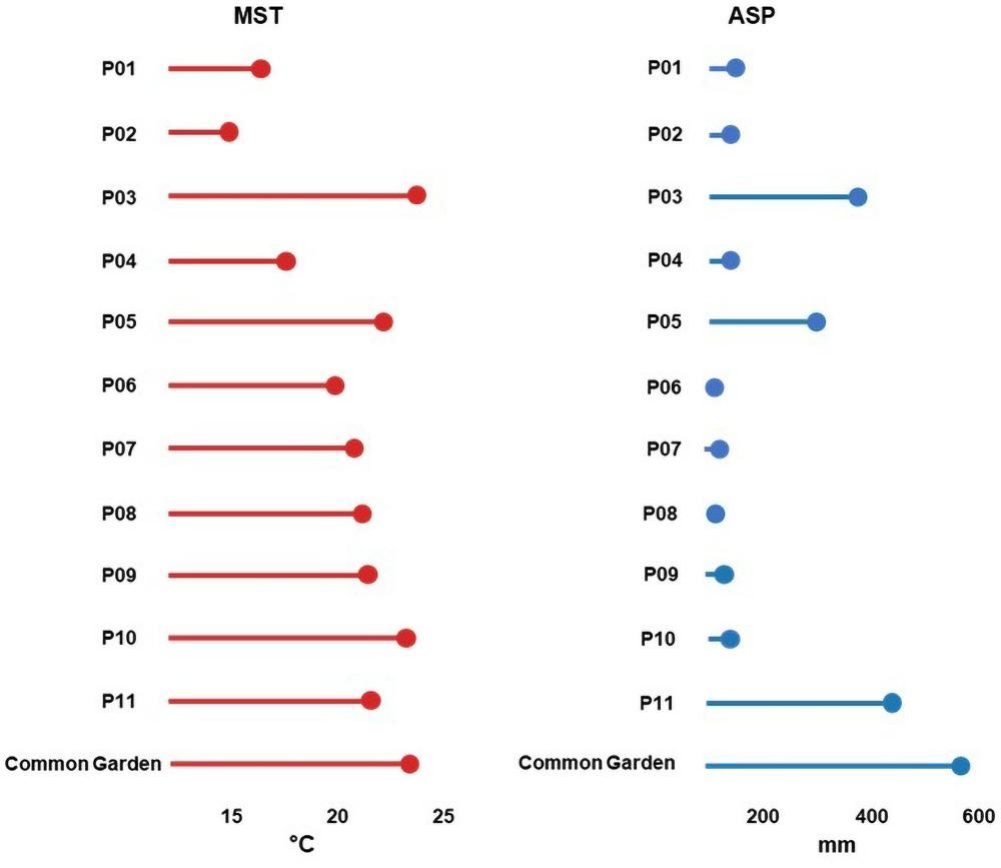
The lollipop plot shows mean summer temperature (left) and accumulated summer precipitation (right) for population origins and the common garden experiment.

This study evaluated the population responses of 11 *Chenopodium hircinum* populations located in the Northernmost extent of the species’ distribution in Argentina. The ecoregions represented by these seed origins are High Monte, Dry Chaco, and Central Andean Puna, in descending order of sample representation (see Supplementary Table S1). Mean Summer Temperature (MST, °C) and Accumulated Summer Precipitation (ASP, mm) data for the population origins during the austral summer months (December–March) were obtained from WorldClim (Harris et al. 2020). We used these two variables (MST and ASP) because the other temperature (e.g. summer maximum and minimum temperatures, annual mean) and precipitation variables (e.g. annual mean precipitation) provided by WorldClim were significantly correlated with each other. Altitude was included since we observed differences among the 11 populations (e.g. Santa Rosa de Tastil vs. Rosario de la Frontera, see Supplementary Table S1). The environmental conditions of the populations, in relation to MST and ASP, are indicated in Fig. 1, including those of the common garden experimental site.

From each population, mature seeds were collected from five individuals. In the lab, the seeds were air-dried at room temperature for 15 days, then cleaned and subjected to a viability test. Four replicates of 25 seeds were sown on 1% agar in Petri dishes and incubated under controlled conditions (25°C with a 12-h light/12-h dark photoperiod) for 30 days. Due to the low initial germination percentage (22 ± 8%), seeds were treated following Curti et al. (2023a). After treatment, germination exceeded 70% in all populations (see Supplementary Table S2). Subsequently, seeds were stored in paper bags in darkness at 15°C until the common garden experiment was conducted. The collected material is preserved in the Native Species Germplasm Bank (BGEN) at INEAH, National University of Salta.

### Common garden experiment and leaf sampling

The common garden is located within the species’ geographic distribution in Northwestern Argentina, and its environmental conditions are similar to those of some of the populations under study (Fig. 1). This experiment was conducted between December 2023 and March of 2024 at the National University of Salta (24.72° S, 65.41° W, 1228 m a.s.l.) under outdoor conditions. Seventy treated seeds per population were sown in 12 cm diametre Petri dishes with 1% agar. One week later, 50 seedlings (with emerged cotyledons) were randomly transplanted to 7-l pots, with five seedlings placed in each pot. Pots were filled with a 3:1 (v/v) mixture of sand and peat substrate arranged in a completely randomized design, with 10 replicate pots per population. To achieve one plant per pot, thinning was performed 10 days post-transplant. The pots were irrigated at dawn prior to recording the physiological variables.

At the anthesis stage, one leaf per plant was harvested from the middle third of the main stem, on which physiological characteristics were previously recorded. Collecting 10 leaves per population was not feasible in any case due to herbivory, limited main stem growth (with early lateral branching), and complete seedling mortality (pre- and post-thinning). Thus, the number of leaves evaluated per population ranged from a minimum of three to a maximum of eight (see Supplementary Table S1). After petiole excision, leaves were positioned adaxially on graph paper and photographed using a digital camera (Nikon D7200, Nikon Corporation Hong Kong) at a consistent distance. Images were saved as standard JPEG files for subsequent image analysis.

### Leaf shape, physiological and functional traits

Shape descriptors, including circularity (CIRC), aspect ratio (AR), and solidity, were computed using ImageJ (Abràmoff et al. 2004) following the conversion of leaf images to binary silhouettes (Curti et al. 2025). Eight previously established landmarks on the leaf outline of *C*. *hircinum* were digitized using the ImageJ point tool (Curti et al. 2025). Additionally, Elliptical Fourier Descriptors (EFDs) were calculated according to the methodology outlined in Curti et al. (2025). Three physiological traits were measured on clear sunny days between 11:00 a.m. and 16:00 p.m.. Measurements included stomatal conductance ($g_{s},\;\;\;\mu \mathrm{mol}\;\;H_{2}O\;\;m^{-2}\;\;s^{-1}$) using a leaf porometer (SC-1 Meter Group, USA), chlorophyll content (CHL, $\mu \mathrm{mol}\;m^{-2}$) using a MC-100 (Apogee Instruments INC, USA), and leaf temperature (LTP, °C) with an infrared thermometer (GM320, China). Three functional traits were evaluated in the same leaves that were used for physiological measurements. Leaf dry mass per unit area (LMA, mg mm^−2^) was determined by weighting leaves (discarding the petiole, LDW mg) and computing leaf area (LA, mm^−2^) from digitalized leaf image.

### Data analysis

All statistical analyses were performed within the R environment (R Core Team 2024). Initial analyses involved a general description of leaf shape based on landmarks and leaf outlines, following a previously published protocol (Curti et al. 2025). Briefly, landmark coordinates were processed using the ‘shapes’ package (Dryden and Mardia 2016) after Generalized Procrustes Analysis (GPA). Then, principal component analysis was applied to the Procrustes-aligned coordinates. Principal component (PC) scores for all retained components (hereafter, Ldk PCs) were determined using the broken-stick model, as implemented in the ‘PCDimension’ package (Wang et al. 2018). The analysis of leaf outlines followed a similar procedure: coordinates of leaf silhouettes were acquired using the ‘Momocs’ package (Bonhomme et al. 2014), and outlines were normalized via a GPA based on the eight defined landmarks. Following alignment, Elliptical Fourier Transforms were fitted separately for the *x* and *y* coordinates. The number of harmonics to be retained for posterior analysis was estimated based on accumulated harmonic power (Bonhomme et al. 2014), and PC scores for the retained components (hereafter, EFD PCs) using the ‘PCDimension’ package.

The relationships among functional and physiological traits (FPTs: LDW, LMA, LA, $g_{s}$, LTP, and CHL), shape descriptors (CIRC, AR, solidity, retained Ldk PCs, and EFD PCs), and environmental conditions (MST, ASP and Altitude) were assessed using Escoufier’s RV coefficients (‘FactoMineR’ package, Josse et al. 2008, Lê et al. 2008) with standardized variables. This statistic quantifies the similarity between the respective configurations, serving as a multivariate generalization of Pearson’s correlation test. A Multivariate Multiple Regression (MMR) analysis (‘car’ package, Fox et al. 2001) was employed to test whether shape and environmental variables significantly predicted FPTs, utilizing a MANOVA (Pillai’s trace statistic). A second MMR analysis was conducted using only the significant predictor variables identified in the first analysis. A comparison between the full and reduced model was performed using an ANOVA. Subsequently, a Multiple Linear Regression (MLR) analysis was run for the best-fitting model in order to identify the predictor variables accounting for variation in each FPTs. Finally, redundancy analysis (RDA) was performed using the ‘vegan’ package (Oksanen et al. 2025). The significance of the model, predictor variables (shape and environmental conditions), and the number of RDA axes were evaluated using a permutation test. A triplot displaying the relationships among the matrices of FPTs, samples, and predictor variables on the retained RDA axes was constructed.

## Results

### Leaf shape variation

**Figure 2.**
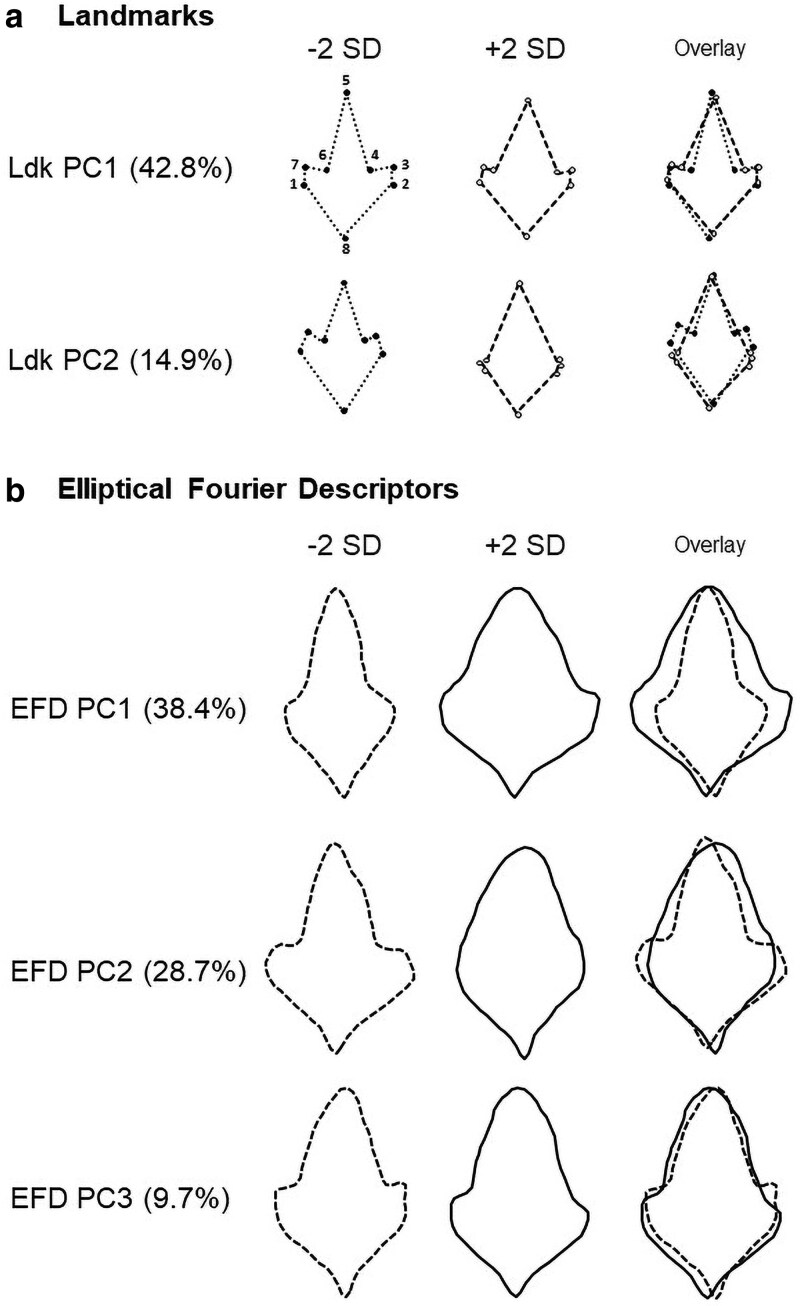
Principal components analyses (PCA) were performed using both (a) landmark coordinates and (b) elliptical Fourier descriptors (EFDs) to generate representative ‘eigenleaves’ summarizing shape variation. For each principal component (PC) that significantly contributed to shape variation, the proportion of explained variance is reported. Shape changes along each PC axis are visualized using reconstructed leaf outlines at ±2 standard deviations (SD) from the mean, solid lines for +2 SD and dashed lines for −2 SD, demonstrating the extent of variation captured. Key shape differences are further emphasized by overlaying these opposing outlines. The positions of eight key landmarks are also indicated.

From the 16 Ldk PCs obtained, only the first two (Ldk PC1 and Ldk PC2) were important to capture significant variation in leaf shape among samples (Fig. 2a). The Ldk PC1 was associated with variations in the relative distances between landmarks 3 and 4, and 6 and 7, distinguishing leaves with pronounced lobulation in the middle section from those with less lobulation in this region (Fig. 2a). In turn, the Ldk PC2 differentiated samples with deeply lobed leaves (trilobed) from those with rounded leaves mainly associated with variation in landmarks 1, 2, 3, 4, 6, and 7 (all of them at the central position along the leaf blade) (Fig. 2a).

**Figure 3.**
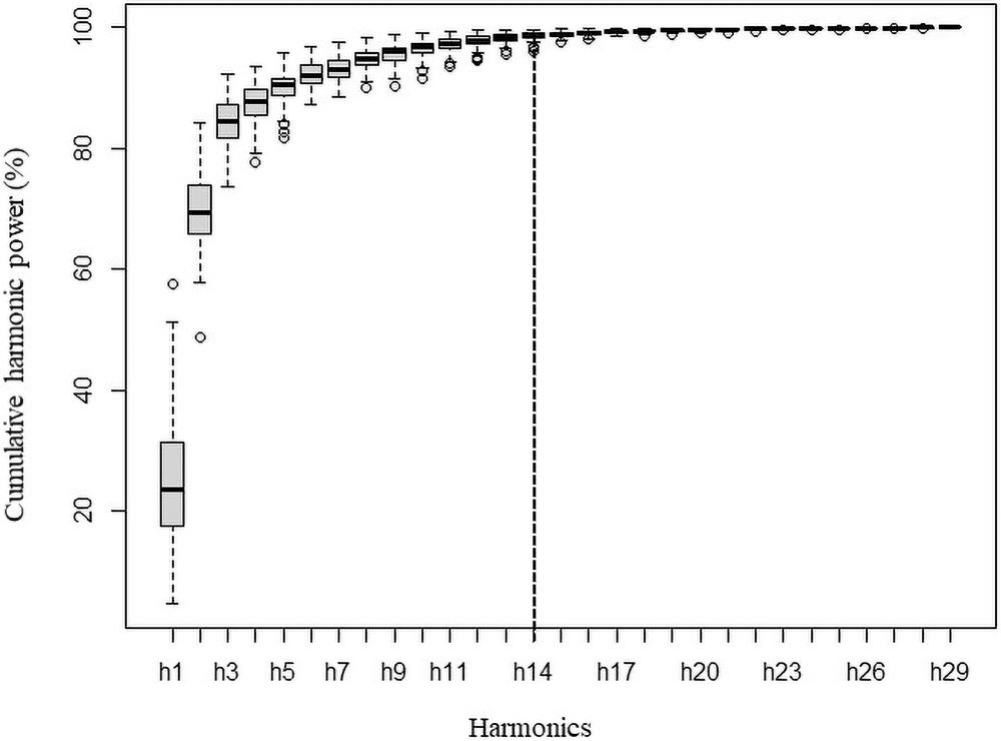
Cumulated harmonic Fourier power calculated from the *C*. *hircinum* leaves. The 14 first harmonics (dashed line) gather nearly 99% of the harmonic power.

Fourteen harmonics were necessary to describe leaf outlines (Fig. 3), whereas only the first three Elliptical Fourier Descriptors PCs captured significant leaf shape variation among samples (Fig. 2b). The EFD PC1 reflects overall leaf outline variations, distinguishing samples with smoother and uniform contours (left) from samples with irregular and wider shapes with an expanded base (Fig. 2b). The EFD PC2 captures changes in leaf area within the middle third, associated with an enlargement of mild-lobing development, differentiating samples with deeply lobulation (trilobed) from samples with rounded leaves (Fig. 2b). On the other hand, EFD PC3 was associated with the development of lobes at the right side in middle-third along leaf blade (Fig. 2b).

### Relationships between leaf shape, environmental conditions and FPTs

A significant association was found between FPTs and shape descriptors, as well as between shape and environmental conditions; however, FPTs showed no direct relationship with environmental variables (Table 1A). A follow-up analysis, separating functional and physiological traits and examining each category of shape descriptors, revealed consistent results. Functional traits were significantly associated with all shape descriptors, whereas physiological traits correlated only with landmark- and Fourier-based measures (Table 1B). Functional and physiological traits were not related. In contrast, all shape descriptors showed significant associations with environmental conditions and with each other (Table 1B).

**Table 1.** Escoufier’s RV coefficients (below diagonal) and their significance (*P*-values, upper diagonal) for the associations between functional and physiological traits (FPTs), environmental conditions (EC) and shape descriptors (ShpD) matrices on a general basis (A) and an individual basis (B).

(A)	FPTs	EC	Shape
FPTs		0.51	1.21×10^−6^
EC	0.04		3.66×10^−4^
Shape	0.26	0.19	

(B)	Funct^a^	Physl^a^	EC	ShpD	Ldks^a^	EFDs^a^
Funct		0.17	0.24	0.01	2.79×10^−7^	1.20×10^−4^
Physl	0.07		0.84	0.11	0.01	0.10
EC	0.02	0.01		3.00×10^−3^	1.00×10^−3^	4.00×10^−3^
ShpD	0.12	0.08	0.15		3.36×10^−8^	4.58×10^−12^
Ldks	0.31	0.12	0.17	0.36		5.97×10^−12^
EFDs	0.19	0.09	0.13	0.45	0.43	

^a^Funct, Functional; Physl, Physiological; Ldks, Landmarks; EFDs, Elliptical Fourier Descriptors.

The full model (MMR analysis) revealed that shape descriptors and environmental variables were significant to predict leaf FPTs (Pillai test statistics = 1.987, approx. *F*_(66, 246)_ = 1.846, *P* = 0.00042). The significant predictors variables were MST, aspect ratio and Ldk PC2 (see Supplementary Table S3). The reduced model (second MMR analysis) considering only MST (Mean Summer Temperature), aspect ratio (AR), and Ldk PC2 predictors variables was also significant (Pillai test statistics = 0.764, approx. *F*_(18,138)_ = 2.622, *P* = 0.00083). The comparison between full and reduced models was significant (Pillai test statistics = 1.41, approx. *F*_(48, 246)_ = 1.574, *P* = 0.014), implying that the reduced model considering only significant predictors was necessary to describe variation in FPTs. The evaluation of the reduced model variables revealed that only AR and Ldk PC2 were significant predictors, both individually and jointly, for all functional and physiological traits, with the exception of CHL (chlorophyll content). The variation in LA (leaf area) alone was accounted for by the joint effect of AR and Ldk PC2 (see Supplementary Table S3).

**Figure 4.**
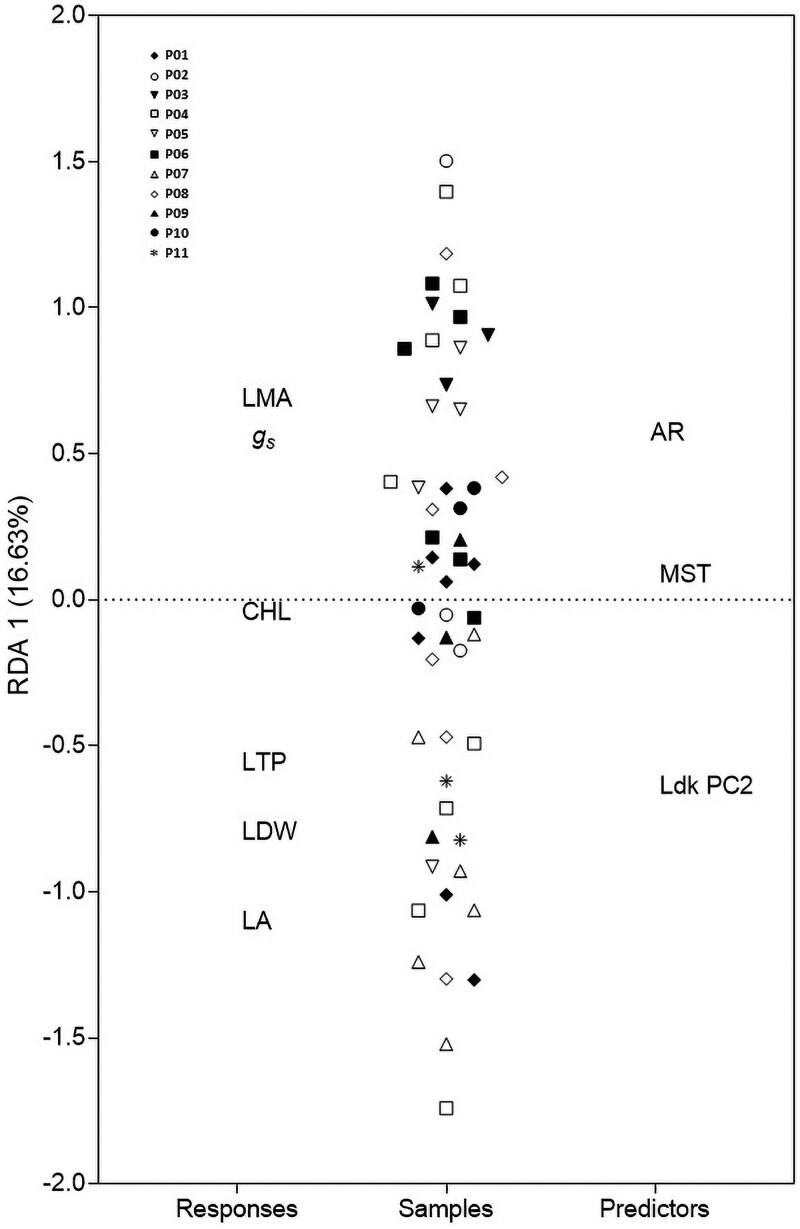
Triplot showing that a significant portion (16.63%) of the variation in leaf traits (LMA, $g_{s}$, CHL, LTP, LDW and LA) can be explained by the environmental (MST) and measured shape predictors (AR and Ldk PC2) on *Chenopodium hircinum* populations (symbols). Interpretation rules are as follows: Variables (Responses and Predictors) placed in close proximity on the plot are positively correlated. Those located on opposite sides indicate a negative correlation. Variables near the dashed line show little correlation with the first RDA axis. Finally, samples positioned near a response or predictor variable have high values for that particular variable.

The RDA showed that shape and climatic variables explain 21% of the variation in FPTs responses. The permutation test confirmed the significance of the MMR reduced model, with only shape descriptors (AR and Ldk PC2) contributing to FPTs variation (see Supplementary Table S4). Only the first RDA component was significant. In the triplot, LMA (leaf mass per area) and $g_{s}$ (stomatal conductance) were positively associated with AR and positioned above the origin, while LA and LTP (leaf temperature) were linked to Ldk PC2 and positioned below (Fig. 4). Samples distributed along the first RDA axis showed strong admixture among populations (Fig. 4). CHL and MST were near the origin, aligning with their low significance and poor predictive capacity for FPTs responses.

## Discussion

This study revealed three main findings: (i) leaf shape variation among *Chenopodium hircinum* populations is significantly correlated with the environmental conditions of their native habitats, at least for the variables evaluated in the present study; (ii) physiological and functional traits were associated only with leaf shape, though independently; and (iii) while a significant amount of the phenotypic variation observed in leaf traits among the sampled populations was genetically determined, individual-level responses ultimately hindered population differentiation and local adaptation. Together, these results highlight a robust relationship among leaf shape, physiological and functional traits, and local climate conditions. This suggests that environmental heterogeneity plays an important role in determining the ecological relevance of leaf shape variation in *C*. *hircinum*.

The observed relationships among leaf shape, FPTs, and environmental condition on a general basis (Table 1A), suggest that leaf morphology may mediate the impact of environmental variation on physiological function. However, while shape descriptors correlated significantly with environmental variables, and shape with FPTs, there were no direct associations between the environment and FTPs alone (Table 1B). Furthermore, although FPTs were both individually correlated with shape, they were not directly correlated with each other (Table 1B). These findings indicate that leaf shape functions as an independent mediator, bridging environmental conditions and plant physiological function. Landmark-based morphometric analysis identified the second principal component (PC2) as the primary axis differentiating deeply trilobed and rounded leaf forms (Fig. 1a). Furthermore, PC2 was also a significant predictor of variation in multiple FTPs (see Supplementary Tables S3 and Fig. 4).

RDA revealed a notable co-localization of leaf mass per unit area (LMA) and stomatal conductance ($g_{s}$) (Fig. 4). Specifically, $g_{s}$ was positively correlated with leaf aspect ratio and negatively correlated with both leaf temperature and lobation (i.e. lower PC2 scores). These results align with theoretical expectations that lanceolate, entire-margined leaves maintain sub-ambient leaf temperatures through enhanced transpiration (Nicotra et al. 2011; Singhal et al. 2025). This occurred as plants lost more water via their stomata when exposed to environments hotter than their native origin (see Fig. 1). Conversely, lobed leaves exhibited higher temperatures and lower stomatal conductance (Fig. 4). While this strategy has been considered adaptive in environments with low water availability (Singhal et al. 2025), our results do not support this hypothesis, as populations from arid and mesic environments displayed a wide range of intrapopulation leaf shape variation associated with differences in physiological responses in concordance with individual responses rather than population ones (Valladares and Sánchez-Gómez 2006).

**Figure 5.**
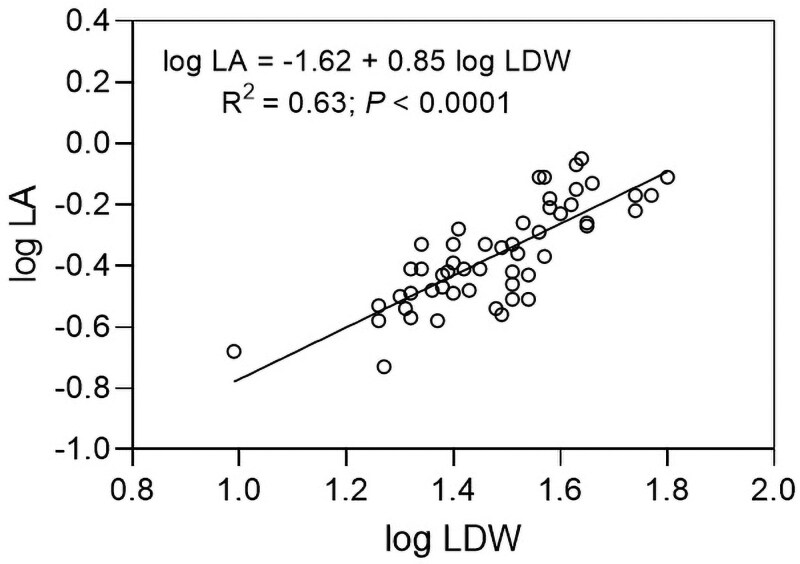
Log–log bivariate relationship for LA vs. LDW. Information about the regression model, coefficient of determination (*R*^2^) and significance (*P*-value) are shown.

According to results, *C*. *hircinum* may adopt a resource-acquisitive strategy given the significantly and positive association found between leaf area and leaf dry weight (Fig. 4). This aligns with global trait patterns (Wright et al. 2004) and as an allometric analysis revealed a slope < 1 between these traits (Fig. 5) was consistent with the principle of diminishing returns in leaf biomass investment (Niklas et al. 2007). However, the positive association found between LMA and $g_{s}$ deviates from the theoretical expectations of the leaf economics spectrum (LES). According to the LES, species employing a resource-acquisitive strategy typically exhibit a low investment of dry mass per unit area, elevated photosynthetic rates and high stomatal conductance. The observed deviation from theoretical expectations could arise because LMA is influenced by both leaf thickness and density, components that can exert contrasting effects on stomatal conductance (Poorter et al. 2009, Shi et al. 2024). Alternatively, while LES outlines global trends, localized environmental conditions—like those in our common garden experiment where populations received increased moisture and irrigation (Fig. 1)—can strongly modulate the LMA-stomatal conductance relationship, leading to a noisier association between these variables (Reich 2014).

A central finding of this study is the broad intraspecific variation in FPTs observed under controlled common garden conditions, indicating a substantial genetic component to trait diversity in *C*. *hircinum*. Such variation, which arises independently of local environmental influences, underscores the value of common garden experiments in uncovering genetically based phenotypic differentiation (Turesson 1922, Scheepens and Stöcklin 2013, Møller et al. 2023). However, more fully disentangling the roles of genetic differentiation and phenotypic plasticity will require future work to incorporate reciprocal transplant experiments or multi-year studies across contrasting environments (de Villemereuil et al. 2018), alongside using seeds multiplied from genetically distinct mother plants to isolate their contributions. Notably, while functional and physiological traits were uncorrelated, both exhibited complex interrelationships mediated by leaf shape, a trait that appears central to coordinating phenotypic responses across the species’ climatically heterogeneous range (Curti et al. 2023a, 2023b, 2025). The predominance of trait variation at the individual rather than population level—mirroring patterns seen in other wild taxa (Westerband et al. 2021, Chandler and Travers 2024), though not universally (Matesanz et al. 2020)—suggests that intra-population diversity in *C*. *hircinum* may play a pivotal role in climate resilience.

## Supplementary Material

plaf049_Supplementary_Data

## Data Availability

The data underlying this article are available in the article and in its online Supplementary material.
